# Tobacco and the risk of acute leukaemia in adults

**DOI:** 10.1038/sj.bjc.6690833

**Published:** 1999-12

**Authors:** E V Kane, E Roman, R Cartwright, J Parker, G Morgan

**Affiliations:** Leukaemia Research Fund Centre for Clinical Epidemiology at the University of Leeds, 30 Hyde Terrace, Leeds LS2 9LN, UK; Department for Haematology, Algernon Firth Building, University of Leeds, Leeds LS2 9JT, UK

**Keywords:** tobacco, acute leukaemia, epidemiology case-control study

## Abstract

Self-reported smoking histories were collected during face-to-face interviews with 807 patients with acute leukaemia and 1593 age- and sex-matched controls. Individuals who had smoked regularly at some time during their lives were more likely to develop acute leukaemia than those who had never smoked (odds ratio (OR) = 1.2, 95% confidence interval (CI) 1.0–1.4). The association was strongest for current smokers, defined here as smoking 2 years before diagnosis (OR = 1.4, 95% CI 1.1–1.7). With respect to the numbers of years smoked, risk estimates were raised in all groups except those who had smoked for fewer than 10 years. Similarly, the odds ratio decreased as the number of years ‘stopped smoking’ increased, falling to one amongst those who had given up smoking for more than 10 years. No significant linear trends were found, however, with either the numbers of years smoked or the numbers of years stopped smoking, and no significant differences were found between AML and ALL. © 1999 Cancer Research Campaign


*British Journal of Cancer* (1999) **81**(7), 1228–1233
© 1999 Cancer Research Campaign
Article no. bjoc.1999.0833

### Tobacco and the risk of acute leukaemia in adults

**EV Kane[1], E Roman[1], R Cartwright[1], J Parker[1] and G Morgan[2]**

[1]Leukaemia Research Fund Centre for Clinical Epidemiology at the University of Leeds, 30 Hyde Terrace, Leeds LS2 9LN, UK; [2]Department for Haematology, Algernon Firth Building, University of Leeds, Leeds LS2 9JT, UK

**Summary** Self-reported smoking histories were collected during face-to-face interviews with 807 patients with acute leukaemia and 1593 age- and sex-matched controls. Individuals who had smoked regularly at some time during their lives were more likely to develop acute leukaemia than those who had never smoked (odds ratio (OR) = 1.2, 95% confidence interval (CI) 1.0–1.4). The association was strongest for current smokers, defined here as smoking 2 years before diagnosis (OR = 1.4, 95% CI 1.1–1.7). With respect to the numbers of years smoked, risk estimates were raised in all groups except those who had smoked for fewer than 10 years. Similarly, the odds ratio decreased as the number of years 'stopped smoking' increased, falling to one amongst those who had given up smoking for more than 10 years. No significant linear trends were found, however, with either the numbers of years smoked or the numbers of years stopped smoking, and no significant differences were found between AML and ALL. © 1999 Cancer Research Campaign

**Keywords:** tobacco; acute leukaemia; epidemiology case-control study

Although little is known of the causes of acute leukaemia in adults, generally accepted risk factors include ionizing radiation, benzene and cytotoxic therapy. Because tobacco smoke contains benzene and radioactive compounds, as well as nitrosamines and urethanes, which have been found to be leukaemogenic in animal experiments (Vesselinovitch and Mihailovich, 1966; Shisa et al, 1975), the hypothesis that tobacco is associated with leukaemia seems plausible. In their 1986 review, Austin and Cole concluded that the epidemiological evidence suggested a weak association between smoking and leukaemia, recommending that future investigations separate lymphoid and myeloid lineages and collect detailed smoking histories. The current report presents the findings for smoking obtained from a large population-based case–control study of acute myeloid and acute lymphoblastic leukaemia.

### MATERIALS AND METHODS

The Leukaemia Research Fund's (LRF) case–control study of adult acute leukaemia ran from 1 April 1991 to 31 December 1996 in the English regional health authorities of South West, Wessex and Yorkshire, and the counties of Lancashire and Cumbria. Cases were all persons newly diagnosed with acute leukaemia during the study period, aged between 16 and 69 years old, and normally resident in the study area. Ascertainment was through weekly or monthly visits by trained investigators to haematological departments of all hospitals in, and on the peripheries, of the study area. Diagnoses were confirmed pathologically. Patients were ineligible if, prior to their diagnosis of acute leukaemia, a definite diagnosis of chronic myeloid leukaemia or myelodysplastic syndrome had been made in the previous 6 months, if they had had any other malignancy in the previous 2 years, or if they had Fanconi's anaemia. Cases were also excluded if they had lived abroad for 6 months or more in the year prior to diagnosis. Following consent from the treating consultant, eligible patients were asked to participate in the study. Cases were usually contacted within 2 weeks of diagnosis or during the first remission (between 4 and 6 weeks after diagnosis). If the patient died before an interview was arranged, their partner or a closerelative was approached, with the permission of the case's treating consultant or general practitioner (GP), between 3 months and a year after death to act as a surrogate. Interviews were not undertaken with any patient who had insufficient command of English, was unable to communicate, or had severe mental disturbance where no suitable surrogate was available.

Two controls per case, individually matched to the case on sex, year of birth, ethnic origin and residence at diagnosis, were randomly selected from general practice lists; the majority being selected from the general practice where the case was registered. If the GP of the case refused, an adjacent general practice was approached. In Somerset, ethical committee permission for control selection from general practice lists was not granted, and controls were randomly selected by the Family Health Services Authority. Following consent from their GP, potential controls were sent a letter asking them to participate. If no reply was received within 2 weeks, an attempt was made to contact them by telephone, and if no reply was received after a month, another control was approached.

A total of 1066 acute leukaemia patients were identified. Interviews were obtained for 838 (79%) cases; 731 (87%) being with the case and 107 (13%) being with a surrogate. Permission to interview was refused by 40 (4%) consultants, 18 (2%) patients and 21 (2%) surrogates. A further 149 (14%) cases were not interviewed, either because a suitable surrogate could not be found, the patient could not be traced, or the subject was interviewed for another study. For the 838 interviewed cases, 3100 controls were asked to participate. Of the controls who were contacted successfully, 1658 (66%) agreed to participate and 854 (34%) refused. It is possible that the majority of the 588 potential controls who could not be contacted would have moved from the address supplied by

*Received 4 January 1999*
*Revised 26 March 1999*
*Accepted 30 March 1999*

*Correspondence to:* EV Kane

**Table 1** Distribution of white interviewed cases and controls by lineage, marital status, age left school, and deprivation

| Variable | Cases *n* (%) | Controls *n* (%) |
|---|---|---|
| Total | 807 (100) | 1593 (100) |
| Diagnosis[a] | | |
| AML | 695 (86) | – |
| ALL | 100 (12) | – |
| Other | 12 (1) | – |
| Marital status | | |
| Single | 138 (17) | 220 (14) |
| Married | 549 (68) | 1154 (72) |
| Divorced/separated | 64 (8) | 116 (7) |
| Widowed | 32 (4) | 66 (4) |
| Other | 24 (3) | 37 (2) |
| Age left school | | |
| ≤16 | 673 (83) | 1277 (80) |
| >16 | 126 (16) | 301 (19) |
| Still at school | 7 (1) | 15 (1) |
| Not known | 1 (0) | 0 (0) |
| Deprivation[b] | | |
| 1 (least deprived) | 135 (17) | 331 (21) |
| 2 | 141 (17) | 290 (18) |
| 3 | 137 (17) | 282 (18) |
| 4 | 114 (14) | 224 (14) |
| 5 | 111 (14) | 197 (12) |
| 6 | 90 (11) | 162 (10) |
| 7 (most deprived) | 76 (9) | 99 (6) |
| Not known | 3 (0) | 8 (1) |

[a]ALL = acute lymphoblastic leukaemia; AML = acute myeloid leukaemia. Other acute leukaemias includes five cases of acute biphenotypic leukaemia and seven cases of unspecified acute leukaemia. [b]Deprivation coded using categories of the Townsend scores for England and Wales; cases are more likely to live in deprived areas than their matched controls (Pearson's $\chi^2$ = 13.67, $P$ = 0.03).

the GP. The present analysis is restricted to 807 Caucasian cases and their 1593 corresponding Caucasian controls; 786 cases have two controls and 21 have only one control.

All participating subjects were asked to complete a pre-interview form, containing details of residential, occupational and medical history. Subjects were subsequently interviewed face-to-face by trained personnel using a highly structured questionnaire. Topics covered included residential history, previous occupations and their exposures, medical history and familial cancer as well as smoking history. Exposure to tobacco was defined as the subject having smoked at least once a day for at least 6 months. Every change in habit, such as the type of tobacco smoked, and the number of cigarettes smoked per day, was recorded with the corresponding start and stop dates. A 2-year latent interval prior to diagnosis is assumed, based on the minimum induction period between initial exposure and the onset of leukaemia observed in cohorts exposed to known leukaemogens (Smith and Doll, 1982; Curtis et al, 1984; Aksoy, 1985; Rinsky et al, 1987).

A deprivation indicator was created using the address at diagnosis. For each address, the postcode was validated against the Post Office Postcode Address File using QuickAddress™ (v2.0) and matched to a 1991 census small area, or 'enumeration district', via the PC2ED program available from Manchester Computing Service (MIDAS). Townsend scores were computed for each enumeration district in England and Wales using data from the 1991 census (Townsend et al, 1988). Categorization of the resulting continuous variable provided a coding scheme for the enumeration districts of the addresses at diagnoses.

Odds ratios (OR) and 95% confidence intervals (CI) for matched analyses were calculated using conditional logistic regression (Breslow and Day, 1980). Tests for trend were conducted using the likelihood-ratio test and the lowest exposure category was defined as the reference group. All analyses were performed using STATA (Stata Corporation, 1997).

### RESULTS

Among white interviewed cases, 695 (86%) were diagnosed with acute myeloid leukaemia (AML), 100 (12%) with acute lymphoblastic leukaemia (ALL) and 12 (1%) had unspecified acute leukaemia (Table 1). A slightly higher proportion of cases than controls were single and had left school before the age of 17 years. Although these differences were not statistically significant, interviewed cases were more likely to live in deprived areas than their corresponding controls ($\chi^2$ = 13.67, $P$ = 0.03). Consequently, deprivation was adjusted for in the subsequent analyses.

Table 2 shows the number of cases and controls by reported smoking status 2 years before diagnosis. For all acute leukaemia combined, 505 (63%) cases and 943 (59%) controls reported that they had regularly smoked tobacco (OR = 1.2, 95% CI 1.0–1.4). Compared with non-smokers, the effect was stronger in current smokers (OR = 1.4, 95% CI 1.1–1.7) than in ex-smokers (OR = 1.0, 95% CI 0.8–1.2). No increased risk was evident among those who smoked for fewer than 10 years (OR = 0.9, 95% CI 0.7–1.2). The odds ratio for those who had smoked for 10–19 years was 1.3 (95% CI 1.0–1.8), with similar risks among those who smoked for 20–29 years, 30–39 years, or 40 or more years, and so no significant linear dose–response relationship was evident.

Although the risk reduced rapidly as the number of years stopped smoking increased, there was no significant linear trend ($\chi^2$ = 0.54, $P$ = 0.76). Further, among smokers, there was no evidence of a dose–response relationship with the number of cigarettes smoked per day at 2 years prior to diagnosis: compared with those who smoked fewer than 15 cigarettes a day, the odds ratios for those smoking 15–24, and 25 or more cigarettes were 1.3 (95% CI 0.7–2.5) and 1.0 (95% CI 0.5–2.1) respectively. The numbers of subjects smoking other types of tobacco were small, but as far as we could tell, the risks were no different to that for cigarette smokers (data not shown).

The majority of cases were diagnosed with AML and the odds ratios for this cell type are generally equivalent to those for all acute leukaemia combined. Although none of the odds ratios for ALL were significantly raised, the point estimates are similar to those for AML.

### DISCUSSION

The main finding is an association between tobacco use and acute leukaemia, which appears to be present for both AML and ALL. The data suggest that the risk is greatest amongst subjects who have smoked for at least 10 years. Further, the risk is elevated among current smokers, and declines to the risk of a non-smoker soon after cessation of smoking. However, no significant dose–response trends were observed, either with number of years smoked or with number of cigarettes smoked per day.

© 1999 Cancer Research Campaign 

**Table 2** Number of cases and controls, adjusted odds ratios[a], and 95% confidence intervals by lineage for smoking status, number of years smoked, and number of years since stopped smoking up to 2 years prior to diagnosis using never smoked as reference

| **Variable** | **Acute leukaemia** | | | | **AML[b]** | | | | **ALL[b]** | | | |
|---|---|---|---|---|---|---|---|---|---|---|---|---|
| | **Case (*n* = 807)** | **Control (*n* = 1593)** | **OR[a]** | **95% CI** | **Case (*n* = 695)** | **Control (*n* = 1374)** | **OR[a]** | **95% CI** | **Case (*n* = 100)** | **Control (*n* = 196)** | **OR[a]** | **95% CI** |
| Smoking status | | | | | | | | | | | | |
| **Never** | **295** | **647** | **1.0** | **–** | **251** | **549** | **1.0** | **–** | **38** | **88** | **1.0** | **–** |
| Ever | 505 | 943 | 1.2 | (1.0–1.4) | 438 | 822 | 1.2 | (1.0–1.4) | 61 | 108 | 1.3 | (0.8–2.2) |
| Not known | 7 | 3 | | | 6 | 6 | | | 1 | 0 | | |
| Current | 295 | 461 | 1.4 | (1.1–1.7) | 257 | 389 | 1.4 | (1.1–1.8) | 37 | 64 | 1.3 | (0.7–2.3) |
| Past | 207 | 472 | 1.0 | (0.8–1.2) | 178 | 424 | 0.9 | (0.7–1.2) | 24 | 43 | 1.3 | (0.7–2.5) |
| Not Known | 3 | 10 | | | 3 | 9 | | | 0 | 1 | | |
| Years smoked[c] | | | | | | | | | | | | |
| <10 | 95 | 219 | 0.9 | (0.7–1.2) | 78 | 179 | 0.9 | (0.7–1.3) | 16 | 37 | 1.0 | (0.5–2.0) |
| 10–19 | 120 | 202 | 1.3 | (1.0–1.8) | 95 | 170 | 1.2 | (0.9–1.7) | 23 | 30 | 2.1 | (0.9–4.7) |
| 20–29 | 103 | 186 | 1.2 | (0.9–1.7) | 94 | 165 | 1.3 | (0.9–1.7) | 8 | 19 | 1.0 | (0.4–2.6) |
| 30–39 | 96 | 177 | 1.2 | (0.9–1.7) | 87 | 156 | 1.3 | (0.9–1.8) | 7 | 16 | 1.0 | (0.4–2.8) |
| 40+ | 82 | 144 | 1.4 | (1.0–2.0) | 75 | 139 | 1.3 | (0.9–1.9) | 7 | 4 | 10.6 | (1.2–90.5) |
| Not known | | 9 | 15 | | 9 | 13 | | | 0 | 2 | | |
| Years stopped[d] | | | | | | | | | | | | |
| 21+ | 54 | 151 | 0.8 | (0.5–1.1) | 47 | 140 | 0.7 | (0.5–1.0) | 4 | 9 | 1.0 | (0.3–4.0) |
| 11–20 | 61 | 133 | 1.0 | (0.7–1.4) | 56 | 120 | 1.0 | (0.7–1.5) | 4 | 10 | 0.9 | (0.2–3.4) |
| 1–10 | 92 | 188 | 1.1 | (0.8–1.4) | 75 | 164 | 1.0 | (0.7–1.4) | 16 | 24 | 1.5 | (0.7–3.1) |
| Current | 295 | 461 | 1.4 | (1.1–1.7) | 257 | 389 | 1.4 | (1.1–1.8) | 37 | 64 | 1.3 | (0.7–2.3) |
| Not known | 3 | 10 | | | 3 | 9 | | | 0 | 1 | | |

[a]Odds ratio adjusted for deprivation, estimated using conditional logistic regression. [b]ALL = acute lymphoblastic leukaemia; AML = acute myeloid leukaemia. [c]Tests for trend in number of years smoked uses <10 years as baseline; $\chi^2 = 0.81$ ($P = 0.85$) for acute leukaemia, $\chi^2 = 0.18$ ($P = 0.98$) for AML, and $\chi^2 = 11.54$ ($P < 0.01$) for ALL. [d]Tests for trend in number of years since stopped uses 21+ years as baseline; $\chi^2 = 0.54$ ($P = 0.76$) for acute leukaemia, $\chi^2 = 1.64$ ($P = 0.44$) for AML, and $\chi^2 = 0.79$ ($P = 0.68$) for ALL.

While published evidence for leukaemia and self-reported tobacco use is inconclusive (Kinlen and Rogot, 1988; Brownson, 1989; McLaughlin, 1989; Garfinkel and Boffetta, 1990; Mills et al, 1990; Spitz et al, 1990; Brownson et al, 1991; Linet et al, 1991; Brown et al, 1992*a*; Sandler et al, 1993; McLaughlin et al, 1995; Engeland et al, 1996; Adami et al, 1998), studies tend to suggest a weak association for AML (Severson, 1987; Brownson, 1989; Severson et al, 1990; Brownson et al, 1991; Brown et al, 1992*a*; Friedman, 1993; Sandler et al, 1993; Mele et al, 1994; Pasqualetti et al, 1997), with only a few studies not reporting an increased risk (Cartwright et al, 1988; Kabat et al, 1988; Spitz et al, 1990; Crane et al, 1992). Indeed, pooling the odds ratios from all case–control studies published to date suggests that smoking could increase the risk of AML by about 20% (Table 3). This increase is similar to those reported in previous meta-analyses (Brownson et al, 1993; Siegel, 1993). However, these estimates should be treated cautiously since there is strong evidence of heterogeneity between studies, possibly arising from heterogeneous populations and/or different study designs (Hardy and Thompson, 1998). Interestingly, the pooled risk estimate changes little when only population-based case–control studies (Severson, 1987; Severson et al, 1990; Brown et al, 1992*a*; Sandler et al, 1993; and the present study) are considered, despite there being less evidence of heterogeneity ($\chi^2 = 4.99$, $P = 0.29$).

Published evidence does not favour an association between ALL and smoking (Kabat et al, 1988; Brown et al, 1992*a*; Sandler et al, 1993; Mele et al, 1994; Pasqualetti et al, 1997), possibly as many studies lack power to detect a significant risk due to small numbers of cases. Indeed, only one case–control study of comparable size to the present has been published, with a risk estimate and 95% confidence interval (OR = 1.3, 95% CI 0.8–2.0) similar to the result reported here (Sandler et al, 1993). This suggests that the increased risk from smoking for ALL may be similar to that for AML.

Dose–response relationships for AML and ALL have previously been evaluated using the number of cigarettes consumed per day (Kabat et al, 1988; Brownson, 1989; Severson et al, 1990; Brownson et al, 1991; Brown et al, 1992*a*; Crane et al, 1992; Friedman, 1993), the number of years smoked (Severson, 1987; Severson et al, 1990; Brown et al, 1992*a*; Friedman, 1993), or pack-years (Severson et al, 1990; Crane et al, 1992; Sandler et al, 1993; Mele et al, 1994). While the majority of studies found no evidence of a trend in risk with number of cigarettes smoked per day (Kabat et al, 1988; Brownson, 1989; Brownson et al, 1991; Brown et al, 1992*a*; Crane et al, 1992), significant increasing trends with increasing number of years smoked were found in two studies (Severson, 1987; Severson et al, 1990). More consistent with this study were the relatively constant odds ratios for AML with years smoked observed in another study (Brown et al, 1992*a*). Although significant increasing trends of pack-years were reported in previous studies (Severson et al, 1990; Sandler et al, 1993; Mele et al, 1994), it was felt that for the current analysis pack-years were inappropriate as not only does this variable not account for time with respect to diagnosis, it also accrues errors resulting from recall bias. Moreover, the presented results suggest that those who stopped smoking over 20 years before diagnosis were not at risk and so an analysis of lifetime exposure expressed in pack-years is probably unsuitable.

Evidence that current smokers have increased risk of acute leukaemia has been found in three other case–control studies (Severson et al, 1990; Brown et al, 1992*a*; Mele et al, 1994).

 *© 1999 Cancer Research Campaign*

**Table 3** Odds ratios for ever smoked cigarettes up to interview with 95% confidence intervals for acute myeloid leukaemia from case–control studies published after Austin and Cole's 1986 review

| Reference<br>Country | Period<br>Age | Cases | Controls | *n* | OR | 95% CI |
|---|---|---|---|---|---|---|
| Kane et al 1999[c]<br>England | 1991–1996<br>16–69 | 807 acute leukaemias | 1593 | 695 | 1.2[a,b] | (1.0–1.4) |
| Pasqualletti et al 1997<br>Italy | 1972–1997<br>16–91 | 1216 haematological malignancies | 1216 | 73 | 2.3[a] | (1.1–4.8) |
| Mele et al 1994<br>Italy | 1986–1990<br>≥30 | 277 leukaemias or refractory anaemia with excess blasts | 467 | 118 | 1.4 | (1.0–1.9) |
| Sandler et al 1993[c]<br>USA | 1986–1989<br>18–79 | 610 acute leukaemias | 618 | 423 | 1.2[b] | (0.9–1.5) |
| Brown et al 1992a[c]<br>USA | 1981–1984<br>≥30 | 578 leukaemias | 1245 | 114 | 1.4[a] | (0.9–2.2) |
| Crane et al 1992<br>USA | 1982–1983<br>≥18 | 60 AML | 60 | 60 | 0.6 | (0.3–1.4) |
| Brownson et al 1991<br>USA | 1984–1990<br>≥20 | 1648 leukaemias | 5138 | 189 | 1.5 | (1.1–2.1) |
| Severson et al 1990<br>USA | 1981–1984<br>20–79 | 106 AML | 128 | 93 | 2.1 | (1.2–3.9) |
| Spitz et al 1990<br>USA | 1985–1988 | 241 leukaemias | 240 | 34 | 0.8 | (0.4–1.5) |
| Brownson 1989<br>USA | 1984–1987<br>≥20 | 725 leukaemias | 2922 | 238 | 1.4[a] | (1.0–1.9) |
| Cartwright et al 1988<br>England | 1979–1986<br>≥15 | 161 AML | 310 | 161 | 0.6 | (0.4–1.0) |
| Kabat et al 1988<br>USA | 1969–1985<br>20–80 | 562 leukaemias | 9342 | 156 | 0.8[a] | (0.6–1.1) |
| Severson 1987[c]<br>USA | 1981–1984<br>20–79 | 114 AML | 133 | 98 | 1.8 | (1.0–3.2) |
| Combined[d] – all studies | | | | | 1.2 | (1.0–1.4) |
| Combined[d] – excluding Kane et al | | | | | 1.2 | (1.0–1.5) |
| Combined[d] – population-based studies | | | | | 1.3 | (1.1–1.4) |

[a]Odds ratios for ever smoked any tobacco. [b]Smoking status lagged by 2 years (Kane et al, 1999) and by 1 year (Sandler et al, 1993) prior to diagnosis. [c]Population-based studies. [d]Test for heterogeneity gives $\chi^2 = 32.61$ ($P < 0.01$) for all studies combined, $\chi^2 = 32.61$ ($P < 0.01$) for all studies except Kane et al 1999, and $\chi^2 = 4.99$ ($P = 0.29$) for population-based studies.

Unlike our report, however, these studies also found associations for ex-smokers. This difference probably reflects the fact that, like others (Smith and Doll, 1982; Curtis et al, 1984; Aksoy, 1985; Rinsky et al, 1987), we lagged the exposure data 2 years before diagnosis, partly to account for latency and partly because of concerns about changing habits as the illness onsets. Hence, individuals who gave up smoking within the 2 years leading up to diagnosis would have been classified as a current smoker in our study and as an ex-smoker (even if given up in the week before diagnosis) in other studies (Severson et al, 1990; Brown et al, 1992*a*; Mele et al, 1994). One of these studies also suggested decreasing risks with increasing number of years since quit smoking, but this result was based on small numbers (Severson et al, 1990).

The level of completeness of cases in this study is known to be high as all cases are registered on the Leukaemia Research Fund's Data Collection Study (Cartwright et al, 1997), which is estimated to be 98.5% complete for AML and ALL (McNally et al, 1997). Of more concern is the high number of non-participating subjects. Cases and controls who refused to participate tended to live in more deprived areas than interviewed subjects, and so were more likely to smoke (Hay and Foster, 1984; Bennett et al, 1996). As the refusal rate for controls is considerably higher than for cases, the resulting risk estimates may be overestimated. However, adjustment for deprivation did not alter the association of tobacco exposure and acute leukaemia (unadjusted OR = 1.2, 95% CI 1.0–1.4).

A major problem with case–control studies of the type reported here is the retrospective collection of self-reported exposure information. As smoking is not a widely accepted cause of leukaemia, exaggeration of exposure by cases seems improbable. While under-reporting by controls cannot be ruled out, the smoking habits reported appear consistent with a sample of the general population (Bennett et al, 1996). For diseases with poor prognosis, a further problem surrounds the issue of surrogate interviews. If they are not attempted, then the completeness of the study is compromised. On the other hand, surrogate information about

*© 1999 Cancer Research Campaign* 

factors such as smoking may be poor. Fortunately, the quick referral of cases into our study ensured that only a small proportion of interviews (13%) were with surrogates. Removing the few proxy interviews made little difference to the risk estimate for ever compared with never smoked (OR = 1.2, 95% CI 1.0–1.5 based on 702 cases and 1385 controls), and the odds ratios for the number of years smoked and the number of years since quit were similarly unaffected (data not shown).

As the effect of smoking on the risk of acute leukaemia appears to be comparatively small, confounding by other factors may be important. Evidence for other risk factors that could be related to smoking is, however, sparse and contradictory. Nonetheless, it is unfortunate that information on habits such as alcohol consumption (Williams and Horm, 1977; Jensen, 1979; Blackwelder et al, 1980; Hinds et al, 1980; Schmidt and Popham, 1981; Carstensen et al, 1990; Brown et al, 1992*b*) and variations in diet (Hursting et al, 1990; Kwiatkowski, 1993) were not collected in this study.

While our increased risk could be confounded by other risk factors, a causal relationship between tobacco smoke and acute leukaemia seems plausible. Known or suspected leukaemogens, for example benzene, radioactive lead and polonium, nitrosamines and urethanes, are present in tobacco smoke. The levels of such chemicals in one packet of 20 cigarettes are considerably less than in some work environments, so it seems unlikely that any one chemical constituent of tobacco smoke could solely cause the increased risk of acute leukaemia. Although the biological mechanisms are unknown, tobacco smoke has been associated with chromosomal defects in the peripheral blood (Perera et al, 1987), immunological changes such as increases in the leucocyte count (Petitti and Kipp, 1986) and decreases in natural killer cell activity (Hersey et al, 1983); all factors that are linked to leukaemia (Heath, 1982). In addition, long-term use exacerbates such changes (Ghosh and Ghosh, 1987; Petitti and Kipp, 1986) while quitting results in a subsequent decline in the leucocyte count (Petitti and Kipp, 1986), an increase in natural killer cell activity (Hersey et al, 1983) and a decrease in DNA damage (Frenzilli et al, 1997). Hence, our observed increased risk of acute leukaemia with 10 or more years of tobacco use and a decline in the odds ratios with increasing number of years since stopping seems reasonable.

In conclusion, AML was found to be associated with smoking, and the risk was greatest for those who smoked for 10 or more years and who were still smoking at 2 years before diagnosis. It appears that the risk of ALL from tobacco was no different to the risk of AML, but small numbers have hampered our investigation. Although no trend was observed for number of years smoked or number of cigarettes per day, there was evidence that the risk of acute leukaemia decreases on cessation of smoking.

### ACKNOWLEDGEMENTS

We thank all consultants, hospital staff, general practitioners, and interviewees who participated in the study. Our thanks also goes to the interviewers: B Pearlman, R Steer, J Antill, A Corrigan, G Demopoulos, M Dickson, M Ewings, J Hodgson, D Hughes, A Johnson, K Kruger, H Lilley, A Linnell, S Muir, J O'Sullivan, S Pope, C Richardson, P Roberts, B Routledge, P Sanders and D Sayer; the computer programmers: I Cope, S Khan and D Rowland; and the clerks: D Bright, B Cooper, H Cusack, S Fitzpatrick, Y Purkis, D Robinson, A Tinkler and R Yarrow. G Dovey, G Law, A Moorman, S Rollinson and A Smith are thanked for their comments on the manuscript.

### REFERENCES

Adami J, Nyrén O, Bergström R, Ekbom A, Engholm G, Englund E and Glimelius B (1998) Smoking and the risk of leukemia, lymphoma, and multiple myeloma (Sweden). *Cancer Causes Control* **9**: 49–56

Aksoy M (1985) Benzene as a leukemogenic and carcinogenic agent. *Am J Ind Med* **8**: 9–20

Austin H and Cole P (1986) Cigarette smoking and leukemia. *J Chron Dis* **39**: 417–421

Bennett N, Jarvis L, Rowlands O, Singleton N and Haselden L (1996) Smoking. In: *Living in Britain: Results from the General Household Survey. Office of Population Censuses and Surveys, Social Survey Division 1994*. HMSO: London

Blackwelder WC, Yano K, Rhoads GG, Kagan A, Gordon T and Palesch Y (1980) Alcohol and mortality: the Honolulu Heart Study. *Am J Med* **68**: 164–169

Breslow NE and Day NE (1980) Classical methods of analysis of matched data. In: *The Analysis of Case–Control Studies*, 6th edn. International Agency for Research in Cancer: Lyon

Brown LM, Gibson R, Blair A, Burmeister LF, Schuman LM, Cantor KP and Fraumeni JFJ (1992*a*) Smoking and risk of leukemia. *Am J Epidemiol* **135**: 763–767

Brown LM, Gibson R, Burmeister LF, Schuman LM, Everett GD and Blair A (1992*b*) Alcohol consumption and risk of leukemia, non-Hodgkin's lymphoma, and multiple myeloma. *Leukemia Res* **16**: 979–984

Brownson RC (1989) Cigarette smoking and risk of leukemia. *J Clin Epidemiol* **42**: 1025–1026

Brownson RC, Chang JC and Davis JR (1991) Cigarette smoking and risk of acute leukemia. *Am J Epidemiol* **134**: 938–941

Brownson RC, Novotny TE and Perry MC (1993) Cigarette smoking and adult leukemia. *Arch Intern Med* **153**: 469–475

Carstensen JM, Bygren LO and Hatschek T (1990) Cancer incidence among Swedish brewery workers. *Int J Cancer* **45**: 393–396

Cartwright RA, Darwin C, McKinney PA, Roberts B, Richards IDG and Bird CC (1988) Acute myeloid leukemia in adults: a case–control study in Yorkshire. *Leukemia* **2**: 687–690

Cartwright RA, McNally RJQ, Rowland DJ and Thomas J (1997) The descriptive epidemiology of leukaemia and related conditions in parts of the United Kingdom 1984–1993. Leukaemia Research Fund: London

Crane MM, Godwin JE, Annegers JF and Keating MJ (1992) Is histological subtype a marker for environmental exposures in acute myelogenous leukemia? *Cancer Epidemiol Biomarkers Prev* **1**: 183–188

Curtis RE, Hankey BF, Myers MH and Young JL (1984) Risk of leukemia associated with the first course of cancer treatment: an analysis of the surveillance, epidemiology, and end results program experience. *J Natl Cancer Inst* **72**: 531–544

Engeland A, Andersen A, Haldorsen T and Tretli S (1996) Smoking habits and risk of cancers other than lung cancer: 28 years' follow-up of 26 000 Norwegian men and women. *Cancer Causes Control* **7**: 497–506

Frenzilli G, Betti C, Davini C, Desideri M, Fornai E, Giannessi L, Maggiorelli F, Paoletti P and Barale R (1997) Evaluation of DNA damage in leukocytes of ex-smokers by single cell gel electrophoresis. *Mutat Res* **375**: 117–123

Friedman GD (1993) Cigarette smoking, leukemia, and multiple myeloma. *Ann Epidemiol* **3**: 425–428

Garfinkel L and Boffetta P (1990) Association between smoking and leukemia in two American Cancer Society prospective studies. *Cancer* **65**: 2356–2360

Ghosh R and Ghosh PK (1987) The effect of tobacco smoking on the frequency of sister chromatid exchanges in human lymphocyte chromosomes. *Cancer Genet Cytogenet* **27**: 15–19

Hardy RJ and Thompson SG (1998) Detecting and describing heterogeneity in meta-analysis. *Stat Med* **17**: 841–856

Hay DR and Foster FH (1984) Intercensal trends in cigarette smoking in New Zealand 2: Social and occupational factors. *NZ Med J* **97**: 395–398

Heath CW (1982) The leukemias. In: *Cancer Epidemiology and Prevention*. WB Saunders: Philadelphia

Hersey P, Prendergast D and Edwards A (1983) Effects of cigarette smoking on the immune system. Follow-up studies in normal subjects after cessation of smoking. *Med J Aust* **2**: 425–429

Hinds MW, Kolonel LN, Lee J and Hirohata T (1980) Associations between cancer incidence and alcohol/cigarette consumption among five ethnic groups in Hawaii. *Br J Cancer* **41**: 929–940

 *© 1999 Cancer Research Campaign*

Hursting SD, Margolin BH and Switzer BR (1990) Diet and human leukemia: an analysis of international data. *Prev Med* **19**: 242–253

Jensen OM (1979) Cancer morbidity and causes of death among Danish brewery workers. *Int J Cancer* **23**: 454–463

Kabat GC, Augustine A and Herbert JR (1988) Smoking and adult leukemia: a case–control study. *J Clin Epidemiol* **41**: 907–914

Kinlen LJ and Rogot E (1988) Leukaemia and smoking habits among United States veterans. *Br Med J* **297**: 657–659

Kwiatkowski A (1993) Dietary and other environmental risk factors in acute leukaemias: a case-control study of 119 patients. *Eur J Cancer Prev* **2**: 139–146

Linet MS, McLaughlin JK, Hsing AW, Wacholder S, Co-Chien HT, Schuman LM, Bjelke E and Blot WJ (1991) Cigarette smoking and leukemia: results from the Lutheran Brotherhood Cohort Study. *Cancer Causes Control* **2**: 413–417

McLaughlin JK (1989) Cigarette smoking and leukemia. *J Natl Cancer Inst* **81**: 262–263

McLaughlin JK, Hrubec Z, Blot WJ and Fraumeni JFJ (1995) Smoking and cancer mortality among US veterans: a 26-year follow-up. *Int J Cancer* **60**: 190–193

McNally RJQ, Rowland D, Roman E and Cartwright RA (1997) Age and sex distributions of hematological malignancies in the UK. *Hematol Oncol* **15**: 173–189

Mele A, Szklo M, Visani G, Stazi MA, Castelli G, Pasquini P, Mandelli F and the Italian Leukemia Study Group (1994) Hair dye use and other risk factors for leukemia and pre-leukemia: a case–control study. *Am J Epidemiol* **139**: 609–619

Mills PK, Newell GR, Beeson WL, Fraser GE and Phillips RL (1990) History of cigarette smoking and risk of leukemia and myeloma: results from the Adventist Health Study. *J Natl Cancer Inst* **82**: 1832–1836

Pasqualetti P, Festuccia V, Acitelli P, Collacciani A, Giusti A and Casale R (1997) Tobacco smoking and risk of haematological malignancies in adults: a case–control study. *Br J Haematol* **97**: 659–662

Perera FP, Santella RM, Brenner D, Poirier MC, Munshi AA, Fischman HK and Van Ryzin J (1987) DNA adducts, protein adducts, and sister chromatid exchange in cigarette smokers and nonsmokers. *J Natl Cancer Inst* **79**: 449–456

Petitti DB and Kipp H (1986) The leukocyte count: associations with intensity of smoking and persistence of effect after quitting. *Am J Epidemiol* **123**: 89–95

Rinsky RA, Smith AB, Hornung R, Filloon TG, Young RJ, Okun AH and Landrigan PJ (1987) Benzene and leukemia. An epidemiologic risk assessment. *N Engl J Med* **316**: 1044–1050

Sandler DP, Shore DL, Anderson JR, Davey FR, Arthur D, Mayer RJ, Silver RT, Weiss RB, Moore JO, Schiffer CA, Wurster-Hill DH, McIntyre OR and Bloomfield CD (1993) Cigarette smoking and risk of acute leukemia: associations with morphology and cytogenetic abnormalities in bone marrow. *J Natl Cancer Inst* **85**: 1994–2003

Schmidt W and Popham RE (1981) The role of drinking and smoking in mortality from cancer and other causes in male alcoholics. *Cancer* **47**: 1031–1041

Severson RK (1987) Cigarette smoking and leukemia. *Cancer* **60**: 141–144

Severson RK, Davis S, Heuser L, Daling JR and Thomas DB (1990) Cigarette smoking and acute nonlymphocytic leukemia. *Am J Epidemiol* **132**: 418–421

Shisa H, Matsudaira Y, Hiai H and Nishizuka Y (1975) Origin of leukemic cells in mouse leukemia induced by *N*-butylnitrosourea. *Gann* **66**: 37–42

Siegel M (1993) Smoking and leukemia: evaluation of a causal hypothesis. *Am J Epidemiol* **138**: 1–9

Smith PG and Doll R (1982) Mortality among patients with ankylosing spondilitis after a single course with X-rays. *Br Med J* **284**: 449–460

Spitz MR, Fueger JJ, Newell GR and Keating MJ (1990) Leukemia and cigarette smoking. *Cancer Causes Control* **1**: 195–196

Stata Corporation (1997) Intercooled Stata 5.0 for Windows 95. Stata Corporation: Texas.

Townsend P, Phillimore P and Beattie A (1988) Health and deprivation: inequality and the North. Croom Helm: London

Vesselinovitch SD and Mihailovich N (1966) Significance of newborn age and dose of urethan in leukemogenesis. *Cancer Res* **26**: 1633–1637

Williams RR and Horm JW (1977) Association of cancer sites with tobacco and

*© 1999 Cancer Research Campaign*
